# Identification of a Biallelic Missense Variant in Gasdermin D (c.823G > C, p.Asp275His) in a Patient of Atypical Gorham‐Stout Disease in a Consanguineous Family

**DOI:** 10.1002/jbm4.10784

**Published:** 2023-06-29

**Authors:** Daniela Tiaki Uehara, Tomoki Muramatsu, Senichi Ishii, Hidetsugu Suzuki, Kazuyuki Fukushima, Yasuhiro Arasaki, Tadayoshi Hayata, Johji Inazawa, Yoichi Ezura

**Affiliations:** ^1^ Department of Molecular Cytogenetics Medical Research Institute, Tokyo Medical and Dental University (TMDU) Tokyo Japan; ^2^ Saku Central Hospital Advanced Care Center Saku Japan; ^3^ Department of Molecular Pharmacology, Graduate School of Pharmaceutical Sciences and Faculty of Pharmaceutical Sciences Tokyo University of Science Chiba Japan; ^4^ Research Core, TMDU Tokyo Japan; ^5^ Department of Joint Surgery and Sports Medicine TMDU Tokyo Japan; ^6^ Department of Occupational Therapy, Faculty of Health and Medical Science Teikyo Heisei University Tokyo Japan; ^7^ Present address: Ome Municipal General Hospital, Ome Tokyo Japan; ^8^ Present address: Department of Orthopedic Surgery Dokkyo Medical University Saitama Japan

**Keywords:** BONE RESORPTION, GASDERMIN D, INFLAMMATION, MACROPHAGE, OSTEOLYSIS

## Abstract

Gorham–Stout disease (GSD), also called vanishing bone disease, is a rare osteolytic disease, frequently associated with lymphangiomatous tissue proliferation. The causative genetic background has not been noted except for a case with a somatic mutation in *KRAS*. However, in the present study, we encountered a case of GSD from a consanguineous family member. Whole‐exome sequencing (WES) analysis focusing on rare recessive variants with zero homozygotes in population databases identified a homozygous missense variant (c.823G > C, p.Asp275His) in gasdermin D (*GSDMD*) in the patient and heterozygous in his unaffected brother. Because this variant affects the Asp275 residue that is involved in proteolytic cleavage by caspase‐11 (as well as ‐4 and ‐5) to generate an activating p30 fragment required for pyroptotic cell death and proinflammation, we confirmed the absence of this cleavage product in peripheral monocytic fractions from the patient. A recent study indicated that a shorter p20 fragment, generated by further cleavage at Asp88, has a cell‐autonomous function to suppress the maturation of osteoclasts to resorb bone matrix. Thus, the present study suggests for the first time the existence of hereditary GSD cases or novel GSD‐like diseases caused by *GSDMD* deficiency. © 2023 The Authors. *JBMR Plus* published by Wiley Periodicals LLC on behalf of American Society for Bone and Mineral Research.

## Graphical abstract

A case of Gorham–Stout disease (GSD, OMIM: 123880) with multiple vanishing bone lesions was identified in a consanguineous family. Whole exome sequencing analysis revealed a homozygous deleterious missense mutation in the caspase cleavage site (Asp275) of the gasdermin D gene (*GSDMD*). The functional importance of this mutation was supported by Western blotting and a recent mouse genetic study.

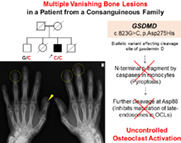

## Introduction

Gorham–Stout disease (GSD), also known as idiopathic massive osteolysis or cystic angiomatosis of bone (OMIM #123880), is an extremely rare disorder characterized by osteolytic lesions with the radiological disappearance of bony contours and is frequently associated with lymphangiomatous tissue proliferation. More than 200 cases have been reported^[^ ^1^, ^2^, ^3^ ^]^ since the first case was described in 1838.^[^ ^4^ ^]^ However, the etiological basis for GSD remains unclear, possibly because of its heterogeneous properties.

A key finding in the diagnosis of GSD is evidence of local and progressive bone resorption with minimal osteoblastic response. The histological coexistence of angiomatous tissue proliferation is not always observed but has been focused on by many researchers.^[^ ^1^, ^5^ ^]^ A differential diagnosis requires ruling out other osteolytic disorders because of different causes, including possible exposure to environmental factors, such as chemical, biological, infectious, immunological, metabolic, and neoplastic causes.^[^ ^5^ ^]^ Other hereditary osteolytic bone disorders also need to be excluded. However, as GSD is essentially unexplained because of its heterogeneous features, the involvement of unknown genetic factors cannot be disregarded.

A recent study suggested that GSDs can be subdivided into types.^[^ ^6^ ^]^ Most patients carrying multiple osteolytic lesions were accompanied by lymphangiomatous tissue proliferation, whereas others without multiple lesions tended not to have such lesions but a history of injury. Because the inflammatory condition is a typical cause of osteolysis, the presence of the latter cases suggests an involvement of the environmental conditions influenced by local tissue inflammation possibly with some intrinsic susceptibility. Although such background may be explained by rare genetic variation(s), this has not been examined before because no previous cases had a familial background suggesting the involvement of hereditary factors.

We recently encountered a case of GSD with multiple vanishing bone lesions. This patient was from a consanguineous family in which the parents were first cousins and were already deceased by the time of his first ascertainment. Because the clinical examinations denied a diagnosis of other already known hereditary bone disorders or metabolic bone diseases, we hypothesized that this patient may represent a novel subtype of GSD harboring a genetic background and thus performed whole‐exome sequencing (WES) on this patient and his oldest living brother.

## Materials and Methods

### Subjects

The patient was a middle‐aged male Japanese office worker aged 54 years on the first visit to our hospital (Saku Central Hospital Advanced Care Center) in 2014. Multiple vanishing bone lesions were detected during his care history, as described in Results, and, thus, a diagnosis of GSD was reached by eliminating the existence of metabolic bone diseases or other known hereditary disorders. An incisional biopsy of the left first fingertip was performed to establish whether lymphangioma vessels were present. A medical interview revealed that the patient belonged to a consanguineous family. Specialized staff for genetics at the Saku Central Hospital Advanced Care Center conducted interviews and created a genealogical family tree. To begin the genetic mutation search, the patient and his unaffected older brother were asked to be involved in a genetic study in 2019.

### DNA samples and ethical statement

Peripheral blood samples were collected from the patient and the brother, and genomic DNA was extracted using standard procedures. Informed consent was obtained from the two individuals. The present study was approved by the Ethical Review Boards at Tokyo Medical and Dental University (approval numbers 02019‐004 and 02021‐006) and the Saku Central Hospital Group Research Ethics Committee (approval number R201908‐11).

### WES

WES was performed on genomic DNA samples of the patient and brother using the Ion Torrent AmpliSeq system (Thermo Fisher Scientific, Waltham, MA, USA). Exome libraries were prepared using the Ion AmpliSeq Exome RDY Kit and sequenced on the Ion Proton platform following the manufacturer's instructions. Base‐calling and read alignments with the human reference genome GRCh37/hg19 were performed using Ion Torrent Suite Software v4.4.0.6. Variant calling was conducted with the Torrent Suite Variant Caller, and Ion Reporter Software was used for the annotation of germline variants. Low‐quality variants were excluded from further analyses.

### Filtering of recessive variants

Because samples from the parents of the patient were not available, the filtering process was initiated with the exclusion of all single‐nucleotide variants (SNVs) with the same genotype in the brother. Only homozygous recessive variants in the patient were selected. The following criteria were then applied to narrow down candidate variants: (i) variants located in the coding region or exon‐intron junctions; (ii) only non‐synonymous variants; (iii) with a minor allele frequency (MAF) <0.001 in population frequency databases, such as the Genome Aggregation Database (gnomAD), 1000 Genomes Project (1KGP), Human Genetic Variation Database (HGVD), and Tohoku Medical Megabank Organization (ToMMo); (iv) with zero homozygotes in the above‐mentioned databases; and (v) estimated as deleterious by predictive tools, such as Polymorphism Phenotyping v2 (PolyPhen2), Sorting Intolerant from Tolerant (SIFT), Combined Annotation Dependent Depletion (CADD), or Rare Exome Variant Ensemble Learner (REVEL).^[^ ^7^ ^]^ Filtered variants were validated by Sanger sequencing.

### RNA extraction, reverse‐transcription polymerase chain reaction (RT‐PCR), and real‐time quantitative RT‐PCR (qRT‐PCR)

Total RNA was isolated from lymphoblastoid cell lines (LCLs) using TRIzol (Thermo Fisher Scientific) and treated with DNase I (Takara Bio, Shiga, Japan). Complementary DNA (cDNA) was synthesized from 1 μg of RNA using the FastGene Scriptase II cDNA kit (NIPPON Genetics Co., Tokyo, Japan). RT‐PCR was performed using two different sets of primers flanking exon 7 of *GSDMD* in which c.823G > C is located. RT‐PCR products were separated by agarose gel electrophoresis and subjected to Sanger sequencing using the BigDye Terminator 3.1 Cycle Sequencing Kit (Applied Biosystems, Carlsbad, CA, USA) and 3730*xl* DNA Analyzer (Applied Biosystems). qRT‐PCR was performed using the KAPA SYBR FAST qPCR Master Mix (KAPA Biosystems, Wilmington, MA, USA) on a 7500 Real‐time PCR System (Applied Biosystems). Primer sequences used in RT‐PCR and qRT‐PCR are available upon request. The RT‐PCR analysis for mouse *Gsdmd* and *Gsdme* was performed as described (Supplemental Fig. S3).

### Human cell culture

The THP‐1 cell line (JCRB0112.1) was obtained from the JCRB Cell Bank (National Institutes of Biomedical Innovation, Health, and Nutrition). LCLs were established from peripheral blood after infection by the Epstein–Barr virus as previously reported.^[^ ^8^ ^]^ These cell lines were cultured in RPMI‐1640 (FUJIFILM Wako Pure Chemical, Osaka, Japan) with 10% fetal bovine serum (FBS). A monocytic fraction of peripheral leukocyte was obtained from the patient and healthy donors using a cell preparation tube with sodium citrate (BD Vacutainer CPT, Beckton, Dickinson and Company, Franklin Lakes, NJ, USA) and was then cultured in Mononuclear Cell Medium (PromoCell, Heidelberg, Germany) without FBS in an ultra‐low attachment six‐well plate. All cell lines and cells were cultured in a humidified atmosphere with 5% CO_2_ at 37°C. THP‐1 cells were authenticated by monitoring their morphology, were routinely checked for Mycoplasma contamination using the TaKaRa PCR Mycoplasma Detection Set (Takara Bio), and were cultured for no more than 20 passages from the validated stocks.

### Mouse cell culture

The RAW264.7 cell line (TIB‐71) was obtained from ATCC and was cultured in α‐MEM (Thermo Fisher Scientific) supplemented with 10% FBS and 1% penicillin–streptomycin (Thermo Fisher Scientific). To induce osteoclast differentiation, RAW264.7 cells were seeded at 7500 cells/cm^2^ on a 12‐well plate (Corning Inc., Corning, NY, USA), and the medium was changed the next day to that containing 100 ng/mL human recombinant receptor activator of NF‐κB ligand (RANKL; PeproTech, Rocky Hill, NJ, USA), followed by a periodic replacement every other day until RNA extraction at each stage. Osteoclast differentiation was also conducted from mouse bone marrow cells as previously described with minor modifications.^[^ ^9^ ^]^ All animal experiments were approved by the Institutional Animal Care and Use Committee of the Tokyo University of Science (approval number Y22039).

### Western blotting analysis of GSDMD cleavage

To generate the inflammasome‐induced cleavage of GSDMD, THP‐1 cells, LCLs, and monocytes from the patient and healthy donors were primed with 1 μg/mL lipopolysaccharide (LPS) (Sigma‐Aldrich Corporation, St. Louis, MO, USA) for 4 hours, followed by a 2‐hour treatment with 10 μM nigericin (Sigma‐Aldrich). Treated cells were then collected for Western blotting analysis. Cells were lysed in Tris buffer (150 mM, pH 6.8) containing 10% sodium dodecyl sulfate (SDS), 15% glycerol, and 10% 2‐mercaptoethanol, and cell lysates were resolved on SDS‐polyacrylamide gel electrophoresis (SDS‐PAGE). The following primary antibodies were used for Western blotting: anti‐GSDMD (ab210070) and anti‐cleaved N‐terminal GSDMD (ab215203) from Abcam (Cambridge, MA, USA) and anti‐β‐actin (A5441) from Sigma‐Aldrich. The signals of bands were detected by FUSION Solo 4S (Vilber, Collégien, France) and SuperSignal West Femto Maximum Sensitivity Substrate (Thermo Fisher Scientific).

## Results

### Clinical findings and history of the patient

**Fig. 1:**
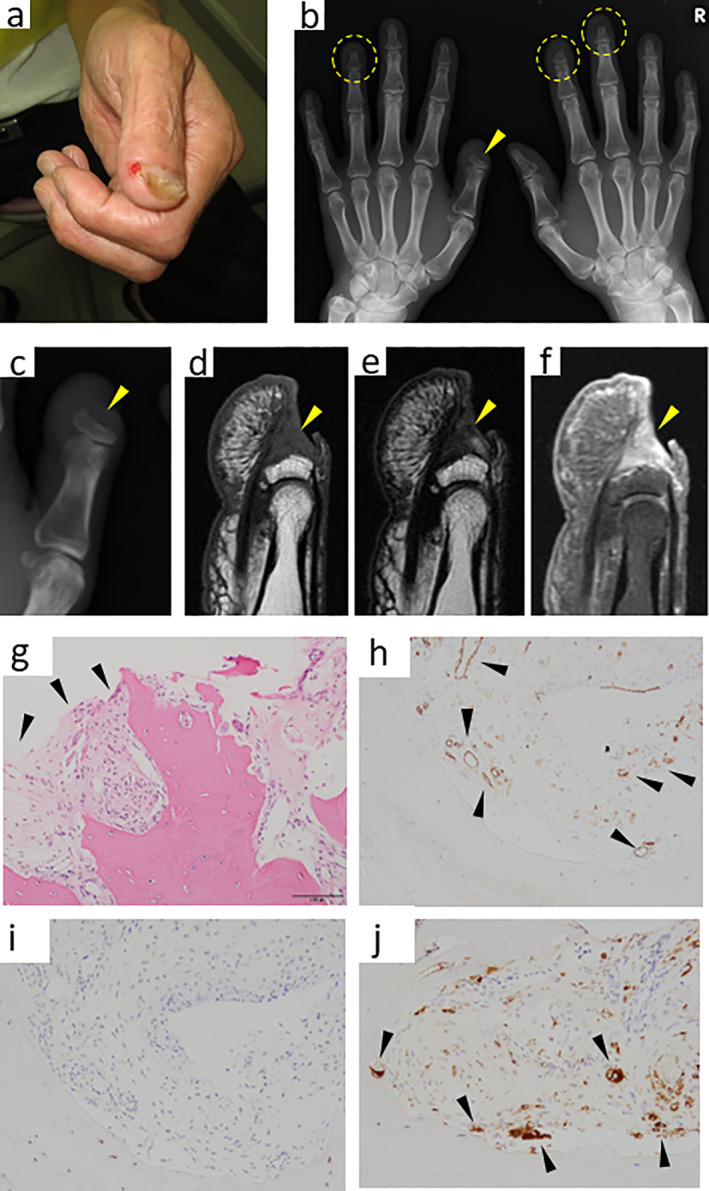
Clinical and pathological findings of the patient's hands. (*A*) Gross appearance of the patient's left thumb. (*B*) Plain radiogram of both hands. The arrowhead indicates the acro‐osteolytic lesion of the left thumb. Dotted circles indicate additional acro‐osteolytic lesions in both hands. (*C*) Magnified plain radiogram of the left thumb (*B*) as a reference for *D*–*F*. (*D*–*F*) Magnetic resonance images (MRI) of the left thumb. The distal phalanx disappeared with the loss of a high‐intensity signal corresponding to bone marrow adipose tissue on T1‐enhanced images (*D*, *E*). Connective tissue remaining on the distal phalanx was enhanced by gadolinium in the T2 image (*F*). (*G*–J) Dissection of biopsy specimens on histology. Standard hematoxylin and eosin showed that the specimen was filled with fibrous connective tissues (arrowheads), blood vessels, and bony fragments (*G*). Blood vessels (arrowheads) were detected by anti‐CD31 immunostaining (*H*). Immunohistochemistry against the D2‐40 antibody (lymphatic vessels) was negative (*I*). Macrophages (arrowheads) were detected by anti‐CD68 immunostaining (*J*). Scale bar = 100 μm.

The proband was a male aged 54 years on his first visit to our hospital in 2014. He had pain in his left thumb for 2 years and had injured that area in 2007 with a forestry tool (machete sickle). Although the wound healed naturally without significant pain, the tip gradually shortened, and pain developed in 2013. A physical examination revealed that the first distal phalanx was visibly shortened, and the lesion was associated with skin erosion and nail deformity, suggesting the presence of chronic paronychia (Fig. 1*A*). A plain radiogram showed marked osteolytic shortening of the distal phalanx of the left thumb (Fig. 1*B*, *C*). In addition, we observed similar osteolytic bone loss in the distal phalanx of the left fourth, right second, and third digits (Fig. 1*B*). Magnetic resonance imaging of the left thumb showed a fibrous or inflammatory granuloma‐like appearance with a low T1 signal and enhanced T2 signal by gadolinium (Fig. 1*D*–*F*). These findings fulfilled the diagnostic criteria of acro‐osteolysis, a type of “vanishing bone” status, on the fingertip that may be caused by heterogeneous disorders, including hormonal alterations, ischemia, traumatic changes, and neurological disorders.^[^ ^10^, ^11^ ^]^ However, in addition to the acro‐osteolysis, we noticed the patient had multiple osteolytic lesions in other parts of the fingers (Fig. 1*B*). For example, multiple subperiosteal osteolytic lesions in the distal metaphyses of the intermediate phalanges were associated with bony defects (3rd and 4th phalanges in the right hand and 2nd phalanx in the left hand), while proximal metaphyses were occupied by small cystlike lesions.

**Fig. 2:**
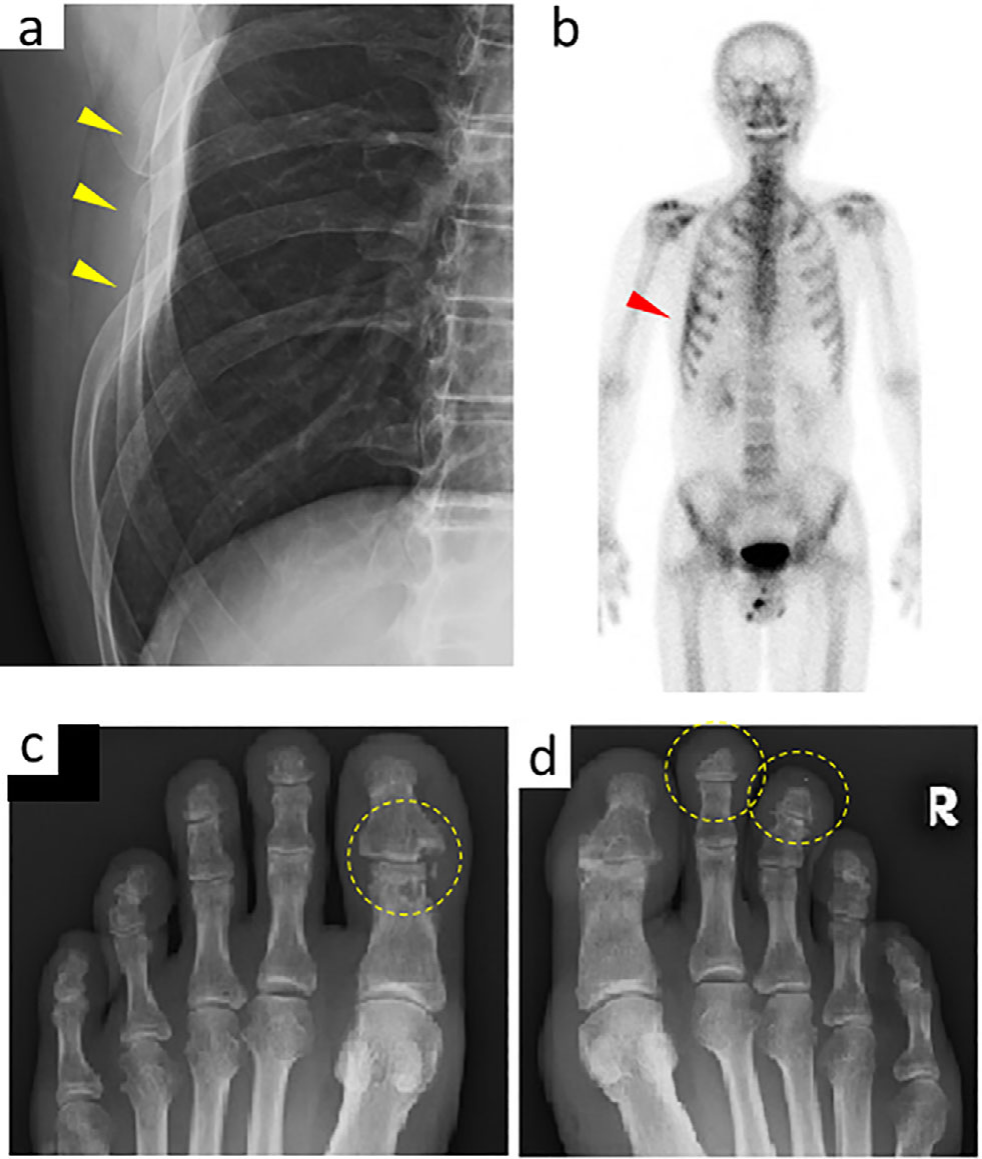
Radiological findings indicate systemic osteolysis in the patient. (*A*) Partial defects in the right ribs (VI–VIII) by a chest radiogram. (*B*) The lateral chest wall on the left side and the thumb of the left hand had hot spots in ^99m^Tc scintigraphy. (*C*) Osteolytic bony defect at the interphalangeal joint of the great toe in the radiogram of the left foot. (*D*) Bony defects on the tips of the second and third toes of the right foot are indicated by dotted circles.

Assuming an involvement of systemic disease in this patient, whole‐body radiograms were taken, and multiple “vanishing bone” lesions were further detected, including prominent defects in the right ribs VI–VIII (Fig. 2*A*). ^99m^Tc radio‐scintigraphy revealed positive spots on the right chest wall as well as the tip of the left thumb (Fig. 2*B*). Furthermore, plain radiographs of the foot showed that the bony contours of the proximal and distal phalanges at the interphalangeal joint partially disappeared in the left great toe (Fig. 2*C*). The second and third toe tips were also affected by shortening (Fig. 2*D*).

Because these findings were consistent with a systemic osteolytic disorder, we checked the data obtained from laboratory examinations on his first visit in 2014. Serum calcium (Ca), phosphate, parathyroid hormone (PTH) levels, serum creatinine, and blood urea nitrogen (BUN) levels were all within normal ranges (Table 1). Clinical interviews and questionnaires denied any possibility of environmental exposure to osteolytic chemicals. Considering the consanguineous background of the patient, we checked for signs of already known hereditary skeletal disorders with systemic osteolytic lesions, including Hajdu‐Cheney syndrome,^[^ ^12^ ^]^ Winchester syndrome,^[^ ^13^ ^]^ and multicentric carpo‐metatarsal osteolysis^[^ ^14^ ^]^ but found none. Therefore, we concluded that GSD was the most probable diagnosis for this case at this time even though an unknown hereditary osteolytic disease was still a possibility. Importantly, despite the above findings suggesting no systemic disease involvement of the patient's skeleton in 2014, the patient subsequently presented with multiple medical conditions, including recurrent bilateral pneumothoraxes, pre‐multiple myeloma with hyper M‐proteinemia, hypertension, hyperlipidemia, and hyperuricemia. Especially, the patient required several hospitalizations to surgically treat spontaneous pneumothorax over the next 9 years of treatment. However, histological findings from the biopsy samples indicated no evidence of lymphangiomatous proliferation, which is known to be frequently associated with GSD. Thus, no apparent association between skeletal lesions and other health conditions was suggested.

**Table 1:** Laboratory Data for the Peripheral Blood Sample from the Patient

		Unit	Normal range	Date
WBC	5900	(/μL)	3300–3800	(June 2014)
RBC	5.00 × 10^6^	(/μL)	4.35–5.55	
Total protein	6.5	(g/dL)	6.6–8.1	
Alubumin	4.1	(g/dL)	4.1–5.1	
ALP	262	(IU/L)	115–330	
Creatinine	0.71	(mg/dL)	0.65–1.07	
BUN	10	(mg/dL)	8–20	
Na	141	(mmol/L)	138–145	
K	3.9	(mmol/L)	3.6–4.8	
Cl	106	(mEq/L)	101–108	
Ca	9.1	(mg/dL)	8.8–10.1	
P	4.3	(mg/dL)	2.7–4.6	(January 2014)
Intact PTH	24	(pg/mL)	14–79	

Abbreviations: ALP = alkaline phosphatase; BUN = blood urea nitrogen; Ca = calcium; Cl = chloride; K = potassium; Na = sodium; P = phosphorus; PTH = parathyroid hormone; RBC = red blood cell; WBC = white blood cell.

**Fig. 3:**
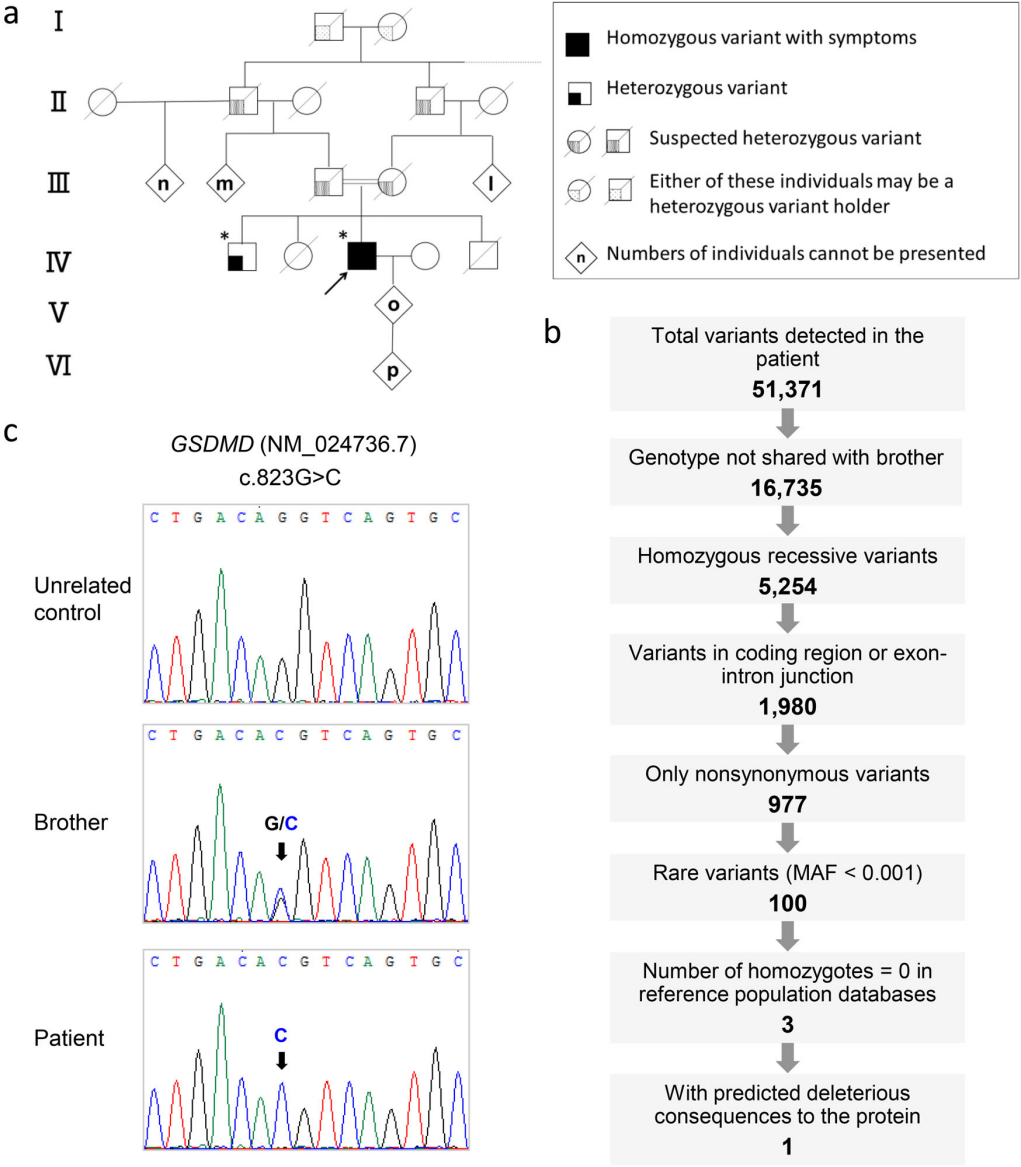
Whole‐exome sequencing (WES) identified a homozygous recessive variant in *GSDMD*. (*A*) A genealogical pedigree tree of the patient's family extending four generations (I–IV). The proband is indicated by an arrow, and asterisks indicate individuals who underwent WES. (*B*) Analysis workflow for filtering rare recessive variants after WES in the patient and his brother. MAF = minor allele frequency. (*C*) Partial Sanger sequencing electropherograms of exon 7 of *GSDMD* (NM_024736.7) in an unrelated control (top), the brother (middle), and the patient (bottom), with an arrow highlighting the recessive variant (c.823G > C, p.Asp275His) detected in the patient. The unaffected brother was heterozygous for the same variant.

The patient was initially treated with an oral bisphosphonate (alendronate; 35 mg/week) but a progressive bone loss at the right fourth fingertip was not prevented. Later, when we administered denosumab (120 mg/4 weeks), it seemed to stop progressive bone loss (Supplemental Fig. S1). However, it was impossible to determine if this was exactly because of the effect of denosumab or just because of the coincidental timing. An incision biopsy was conducted on the left thumb to investigate whether osteolytic lesions included vascular lesions. The timing of the biopsy might have been too late. Standard histology and immunohistochemistry indicated that connective tissue adjacent to the edge of the disappearing distal phalanx was fibrous and vascular components were present (Fig. 1*G*, *H*, *J*). However, lymphangiomatous vessels were not apparent (Fig. 1*I*). The connective tissue surrounding the bone edge also had mild inflammatory cell infiltration mainly with macrophages (Fig. 1*J*). The absence of lymphangiomatous lesions is not a rare event in GSD cases; however, the inconsistency of our findings with the tendency reported by Tanoue and colleagues^[^ ^6^ ^]^ that GSD patients harboring multiple vanishing bone lesions were likely to have lymphangiomatous tissue proliferation may indicate that our case represents a novel type of the GSD or GSD‐like disease. Because the parents of the patient were first cousins (Fig. 3*A*), it was assumed that genetic factors are involved in this patient's characteristically novel diseased status. Thus, we started to search for genetic mutations in this patient and his family members.

### Molecular analysis by WES of the patient and his brother

WES analysis was performed under the premise that the disease follows an autosomal recessive inheritance and is caused by a rare recessive variant. We focused our analysis on homozygous germline variants in the patient that were not present or that were present at least in heterozygosity in the brother. As a result, 51,371 variants were detected in the patient, of which 16,735 had genotypes that differed from those of the brother. By narrowing down candidate genes as shown in Figure 3*B*, three rare recessive variants with zero homozygotes in population databases were detected (Table 2). In consideration of the functional relevance of the gene and the prediction of consequences to the protein, we identified a missense variant (c.823G > C, p.Asp275His) in *GSDMD*, which was present in the brother in the heterozygous state, as the best candidate (Fig. 3*C*).

**Table 2:** Summary of Three Rare Variants With Zero Homozygotes in Population Databases Detected in the Patient After Whole‐Exome Sequencing Analysis

Gene (Ref Seq transcript)	Genomic location (GRCh37/hg19)	dbSNP	Alteration	Prediction of damage to the protein			
				PolyPhen2	SIFT	CADD	REVEL
*FTMT* (NM_177478.2)	chr5:121188110	rs199810128	c.452A > G (p.Asp151Gly)	Benign (0.016)	Damaging (0.03)	22.5	0.267
*SLC45A4* (NM_001286646.2)	chr8:142228487	rs766675251	c.1099G > A (p.Ala367Thr)	Benign (0.005)	Tolerated (0.91)	0.03	0.059
*GSDMD* (NM_024736.7)	chr8:144643998	rs780944198	c.823G > C (p.Asp275His)	Probably damaging (1.000)	Damaging (0.03)	33	0.229

Abbreviations: CADD = Combined Annotation Dependent Depletion; dbSNP = Single‐Nucleotide Polymorphism database; PolyPhen2 = Polymorphism Phenotyping v2; REVEL = Rare Exome Variant Ensemble Learner; SIFT = Sorting Intolerant from Tolerant.

*GSDMD* encodes gasdermin D, a member of pore‐forming effector proteins that is a key regulator of pyroptosis, a type of programmed necrosis that involves pore formation, membrane rupture, and the leakage of cytosolic contents in many cells in the immune system and gastrointestinal epithelial cells.^[^ ^15^, ^16^ ^]^ To date, no variants in *GSDMD* have been associated with any human disease, and only SNVs classified as benign are registered in the ClinVar database. Because the inflammatory responses of macrophages are the most important causes generating osteolytic lesions,^[^ ^17^ ^]^ we considered the candidacy of *GSDMD* p.Asp275His for the causality of our case. A significant deleterious effect of this variant was predicted by several in silico tools (Table 2). Among exome/genome databases, c.823G > C was found in six individuals in the gnomAD data set with a global MAF of 0.00002465, all in heterozygosity. Notably, all six individuals were East Asians. Moreover, in the largest variant database of the general Japanese population, composed of the genotype frequency of approximately 38,000 individuals (ToMMo 38KJPN), the variant was found in 27 individuals with a MAF of 0.00034865. Importantly, no homozygotes were described in either database.

### Molecular analysis of *GSDMD* gene expression

The *GSDMD* c.823G > C variant is located at the exon‐intron splice junction of exon 7 (RefSeq NM_024736.7) (Supplemental Fig. S2 *A*), and, thus, we initially suspected that this variant may affect splicing and consequently reduce GSDMD mRNA expression (in the case of intron retention causing a frameshift) or produce a shorter protein (in the case of exon skipping leading to an inframe deletion). An analysis using Human Splicing Finder suggested that this variant causes alterations in the wild‐type donor site. To investigate this, we performed RT‐PCR and qRT‐PCR using cDNA extracted from the LCLs of the patient; however, no differences from the controls were observed (Supplemental Fig. S2 *B*–*D*). We concluded that c.823G > C did not appear to alter splicing, and, thus, GSDMD protein levels were not expected to be decreased or GSDMD to be shorter in the patient.

We also investigated whether *GSDMD* was functionally expressed in osteoclasts or macrophages in cultures. To achieve this, the macrophagic mouse cell line RAW264.7 was cultured to induce osteoclast differentiation using RANKL. The qRT‐PCR analysis showed that the expression of *Gsdmd* (the mouse ortholog of *GSDMD*) gradually increased during osteoclast differentiation, whereas the expression of *Gsdme*, a closely related member of the GSDM family, decreased (Supplemental Fig. S3 *A*, *B*). The observation was consistent with a recent study by Xiao and colleagues^[^ ^18^ ^]^ and that by Aki and colleagues, using the same cell line RAW264.7 without osteoclast induction.^[^ ^19^ ^]^ Osteoclasts were also generated from mouse bone marrow cells by induction using macrophage colony‐stimulating factor (M‐CSF) and RANKL to examine expression. The qRT‐PCR analysis similarly indicated a gradual increase in the expression of *Gsdmd* during the culture, whereas that of *Gsdme* decreased (Supplemental Fig. S3 *C*, *D*).

### Molecular support for the findings

**Fig. 4:**
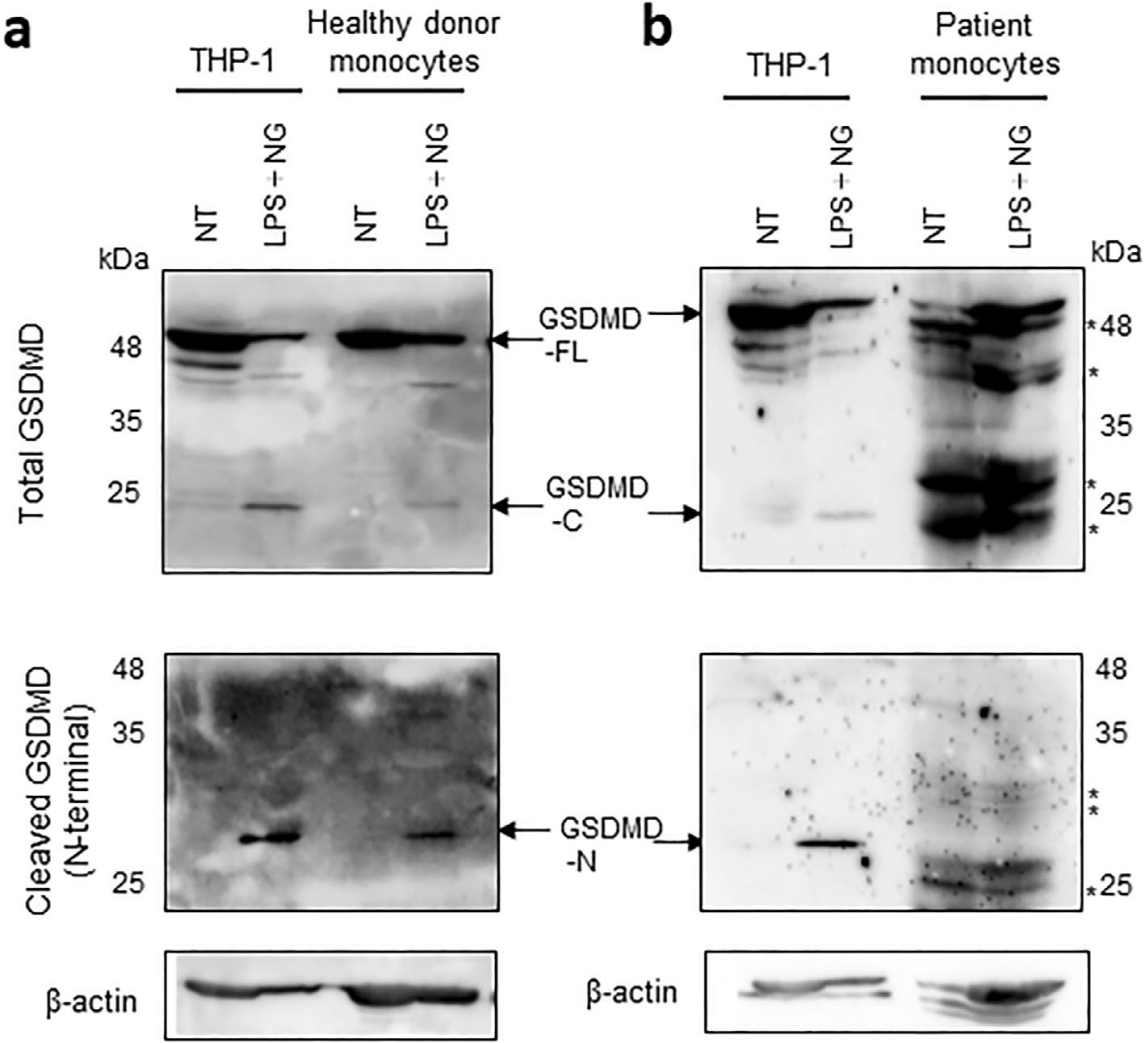
GSDMD p.Asp275His resists cleavage in patient monocytes. (*A*) Western blotting analysis of total GSDMD (top) and cleaved GSDMD (bottom) was performed using THP‐1 cells and the monocyte fraction from the peripheral leukocytes of a healthy donor. The cleaved ~31‐kDa N‐terminal fragment of GSDMD (GSDMD‐N) was detected in all samples. The cleaved ~24‐kDa C‐terminal fragment was also detected by the ab210070 total antibody. (*B*) A Western blotting analysis of total GSDMD (top) and cleaved GSDMD (bottom) was performed using THP‐1 cells and the monocyte fraction from the peripheral leukocytes of the patient. No cleavage of GSDMD was detected in patient monocytes after LPS priming and nigericin stimulation, even after prolonged exposure with the appearance of numerous nonspecific bands (indicated by asterisks). The appearance of numerous nonspecific bands in the patient's lysate may be due to the influence of the prolonged time required for transferring the patient's blood from a remote location. GSDMD‐C = C‐terminal GSDMD; GSDMD‐FL = full‐length GSDMD; GSDMD‐N = N‐terminal GSDMD; LPS = lipopolysaccharide; NG = nigericin; NT = no treatment. *Nonspecific bands.

The inflammatory activation of monocytic and macrophagic cells may induce systemic osteolysis through inflammasome activation and cytokine release. Previous studies indicated that caspases (‐1, ‐4, ‐5, and ‐11) activated by invasive pathogens and danger signals cleaved GSDMD to enhance cytokine release and trigger pyroptotic cell death.^[^ ^20^ ^]^ In human GSDMD, the substrate recognition motif is a conserved _272_FLTD_275_ sequence and cleavage by caspases occurs after Asp275,^[^ ^15^ ^]^ which was the residue mutated in our patient. This prompted us to speculate that the abrogation of the cleavage site caused by the homozygous recessive variant may affect recognition by caspases and consequently impair pyroptosis in patient cells upon pathogen infection. Therefore, to establish whether pathogen‐induced cleavage was defective in the mutant GSDMD p.Asp275His, we used the established LCLs from the patient and control subjects at first. However, because it was not effective to see the cleaved fragments of GSDMD from the extracted lysate from these cell lines, we tried to use a monocyte fraction obtained from the peripheral leukocytes of the patient (Supplemental Fig. S4). When using the human monocytic cell line THP‐1 as a positive control, the Western blotting analysis showed a consistent N‐terminal fragment at 31 kDa and a C‐terminal 24‐kDa fragment of GSDMD after LPS priming and a nigericin treatment (Fig. 4*A*). Similarly, the monocyte fraction from healthy controls consistently showed a cleaved N‐terminal fragment after treatment with LPS and nigericin (Fig. 4*A*). However, the monocyte fraction from the patient did not show these fragments (Fig. 4*B*), suggesting failed activation by cleavage. We noticed our finding was consistent with a previous report analyzing the functional outcomes by cell culture experiments of various human *GSDMD* polymorphisms predicted to be deleterious.^[^ ^21^ ^]^

To conduct a more functional in vivo study, we aimed to redo the experiment with additional blood sampling. However, our patient died of acute ischemic heart disease in November 2022, with multiple health problems, including recurrent pneumothorax, COPD, idiopathic hypertension, pulmonary hypertension, hyperlipidemia, alcoholic hepatitis, and IgGκ‐type M proteinemia, suggesting early stages of multiple myeloma. On the other hand, during the preparation of this article, critical findings that support osteolytic symptoms because of the deficient GSDMD were reported by Li and colleagues.^[^ ^22^ ^]^ They showed functionally that the mice deficient for *Gsdmd* lose bone mass because of the activated osteoclast bone resorption through a novel cell‐autonomous pathway through the N‐terminal cleaved fragment p20 by caspases in the osteoclasts.^[^ ^22^ ^]^ Therefore, functional bases for the bone loss observed in our patient could be explained by enhanced osteoclast activation because of the loss of the p20 fragment of GSDMD.

The discovery of a probable causative mutation in *GSDMD* in our GSD patient also requires support from additional GSD patients with similar mutations in *GSDMD* or with mutations in functionally related genes. However, because of the rarity of this disease, no additional cases were obtained for genetic analysis in the present study. Information obtained through personal communications is instead described in Discussion.

## Discussion

We herein report an extremely rare case of GSD that may harbor a genetic background, which is entirely novel. We detected this *GSDMD* variant p.Asp275His in our case in early 2021. In the gnomAD database, this variant was only found in the East Asian population at an extremely low frequency (0.0003), being absent from other population data sets. In addition to its rarity, the possible causality of the mutation is supported by in silico prediction tools and the expression of GSDMD in monocyte/macrophage lineage cells (Supplemental Fig. S3).^[^ ^19^, ^20^ ^]^ Because there have been no case reports of GSD associated with causative germline mutations in the literature, we appear to have discovered a novel aspect of this osteolytic disorder.

The p.Asp275His alteration identified in *GSDMD* was refractory to cleavage by caspase‐11 in monocytic leukocytes through activation by infectious pathogens, and importantly, p.Asp275His was reported on the pathway related to inflammasome activation. Since the involvement of inflammatory cell death with cytokine release has long been an interesting topic in osteolytic disorders, the mutation resulting in the impairment of cleavage was initially thought to be paradoxical to be associated with osteolysis in the present case. If the macrophages are deficient in cleavage after the LPS stimulation, this was expected to result in a loss of pyroptotic cell death after stimulation by pathogens, for example, and this may lead to an insufficient inflammatory response of the macrophages to the infectious agents with reduced cytokine release. If we were to think simply, the resulting symptoms should be osteosclerosis, not osteolysis.

When we started to search for literature information about pyroptotic cell death and GSDMD, we could not obtain enough information about its possible influence on the skeletal system. However, later, we noted that one research group had started to focus on *GSDMD* as a contributing factor to bone remodeling and inflammatory bone loss,^[^ ^20^, ^23^ ^]^ and this was followed by another very important study from a different group that indicated a novel cell‐autonomous function of GSDMD by caspase‐8 cleavage in the osteoclasts.^[^ ^22^ ^]^ The authors showed that the cleavage at Asp88 in addition to Asp275 leads to the accumulation of the shorter N‐terminal p20 fragment in the cytosol, resulting in suppression of the enhanced activation of osteoclasts by RANK signaling. Based on this theory, we could suppose the loss of cleavage at Asp275 can result in the overactivation of osteoclastic bone resorption, which may contribute to the formation of vanishing bone lesions in our case. Therefore, our discovery has an important impact by suggesting an example of the etiological involvement of this novel function of GSDMD in vanishing bone lesions or osteolysis in humans for the first time. Osteolytic lesions of GSD sometimes occur after a traumatic event but usually occur spontaneously with unknown triggers. There is some preference in the location of the lesions in the trunk and head, and the occurrence in extremities is less frequent. Therefore, our case was somehow different from the usual cases in the literature on this point too. Further analyses both in vivo and in vitro about the GSDMD‐medicated bone loss are required.

The identification of similar cases with *GSDMD* mutations is important to indicate genotype–phenotype correlations. We first tried to search for such cases in our research community. However, the extreme rarity of the disease makes it difficult to conduct in a single institute. We then asked other investigators who reported GSD cases previously for searching pathogenic or candidate variants in GSDMD (or other related genes). However, in nine Japanese patients with GSD who were examined, no such variants were identified (Ozeki M and Aoki Y, personal communications). We believe if many of the internationally available GSD cases were examined for genetic mutations, similar cases may be found in the future. Moreover, the significance of the associated symptoms in other organs, including pneumothorax, hematologic malignancies, and ischemic heart disease, may become clear if multiple cases are found in the future. Such a mutation search may need to be performed on a global basis; however, since *GSDMD* p.Asp275His was only detected in East Asians in the gnomAD database, other GSD cases with *GSDMD* mutations are more likely to be found in East Asia rather than in other areas of the world. It currently remains unclear whether biallelic variants affecting other residues have a similar deleterious impact to p.Asp275His.

As our study suggests an existence of a novel subtype of GSD or a novel congenital disorder resembling GSD, the clinical feature of our present case must be confirmed. The diagnosis of our GSD case was based on the presence of multiple vanishing bone lesions that had progressed over several years. A differential diagnosis also ruled out other recessively inherited osteolytic disorders. The absence of evidence for lymphangiomatous proliferative lesions like in many cases in literature may indicate the presence of different subtypes of GSD or GSD‐like disease. Regarding the osteolytic lesion of the left thumb, the patient had a history of mild trauma before the occurrence of one of the vanishing bone lesions, which is consistent with many cases reported in the literature.^[^ ^1^, ^6^ ^]^ A proposal for the classification of GSD has just started. In 2018, in a comparison of 76 cases of GSD in the literature, Tanoue and colleagues suggested the presence of two types of GSD, namely, primary multifocal GSD and trauma‐induced secondary GSD.^[^ ^6^ ^]^ They showed that cases of primary GSD were diagnosed at 14.3 ± 13.2 years,^[^ ^6^ ^]^ with multifocal vanishing bone lesions that were sometimes associated with lymphangiomatous proliferating lesions, whereas secondary GSD cases were mostly diagnosed at 29.6 ± 18.8 years and patients characteristically had a history of trauma for vanishing bone lesions. Therefore, the present case represented a combination of the clinical features of both GSD types, implying the existence of another group of GSD representing multifocal vanishing bone lesions that may be induced by minor trauma, but with the absence of lymphangiomatous lesions and delayed onset. Furthermore, in consideration of the possibility of a causative genetic mutation in our case, a real classification of GSD may be more complex, including cases associated with a single causative gene mutation, or cases associated with somatic mutations for the lymphangiomatous tumorlike lesions.^[^ ^24^ ^]^

In summary, we encountered an extremely rare case of multiple vanishing bone lesions in a consanguineous family with a biallelic missense mutation affecting a critical residue on *GSDMD*. The involvement of *GSDMD* variants in human disease has not been previously recognized. Therefore, our study highlighted that GSD is a complex heterogeneous disorder with different causes, and it includes monogenic cases with a mutation in *GSDMD*. Our discovery may open a novel field of investigation on osteolytic disorders, linking them to a novel molecular mechanism of regulation in osteoclast activation.

## Author Contributions

**Daniela Tiaki Uehara:** Data curation; formal analysis; investigation; methodology; writing – original draft. **Tomoki Muramatsu:** Formal analysis; methodology. **Senichi Ishii:** Conceptualization; data curation. **Hidetsugu Suzuki:** Data curation. **Kazuyuki Fukushima:** Supervision. **Yasuhiro Arasaki:** Data curation; formal analysis. **Tadayoshi Hayata:** Investigation; methodology; validation. **Johji Inazawa:** Project administration; supervision; validation. **Yoichi Ezura:** Conceptualization; funding acquisition; investigation; project administration; writing – review and editing.

## Conflicts of Interest

All authors declare no competing financial interests. The funders had no role in the design of the study, in the collection, analyses, or interpretation of data, in the writing of the manuscript, or in the decision to publish the results.

### Peer Review

The peer review history for this article is available at https://www.webofscience.com/api/gateway/wos/peer‐review/10.1002/jbm4.10784.

## Supporting information


**Fig. S1.** Clinical outcome with time‐course changes in the osteolytic defect of the fourth distal phalanx of the right hand indicated by plain radiograms (*A*–*H*). Radiograms were captured at the first visit in 2014 (*A*, *C*), 1 year later (*D*), 2 years later (*E*), 3 years later when the denosumab treatment was started (*F*), and 1 and 2 years after the denosumab treatment (*G*, *H*, respectively).
**Fig. S2.** (*A*) Schematic representation of *GSDMD* variant 1 (NM_024736.7). Light gray boxes represent coding exons, whereas dark gray boxes represent untranslated regions. The red arrow points to the position of c.823G > C in the exon‐intron junction of exon 7. The black arrows depict the two primer sets used for RT‐PCR. F = forward; R = reverse. (*B*) RT‐PCR in cDNAs derived from the LCLs of the patient and two controls using two different sets of primers flanking exon 7 of *GSDMD*. If c.823G > C alters splicing in patient LCLs, a product that is 87 bp shorter may represent the skipping of exon 7, whereas a product that is 130 bp longer indicates the retention of intron 7. Nevertheless, an RT‐PCR product with the same size as those in controls was observed. (*C*) Partial electropherograms of the Sanger sequencing of RT‐PCR products using primer pair 1 confirmed no skipping of exon 7 or the retention of intron 7 in the patient (bottom), similar to control (top). The position of the c.823G > C variant is highlighted with a rectangle box. (*D*) qRT‐PCR of GSDMD performed in the patient and three controls showed no significant differences in mRNA levels.
**Fig. S3.** (*A*, *B*) qRT‐PCR of mouse *Gsdmd* (*A*) and *Gsdme* (*B*) was performed in an osteoclast‐inducing culture from RAW 264.7 cells. Relative expression normalized against *Gapdh* was standardized against the value on day 0. (*C*, *D*) RT‐PCR of mouse *Gsdmd* (*C*) and *Gsdme* (*D*) was performed in an osteoclast‐inducing culture from bone marrow–derived macrophages. Relative expression normalized against *Gapdh* was further standardized against the value on day 0. Data are expressed as the mean ± SEM; ns: *p* > 0.05, *p < 0.05, **p < 0.01 by a two‐way ANOVA with Šídák's post hoc tests. Statistical analyses were performed using GraphPad Prism 9.
**Fig. S4.** Monocytic fractions of peripheral blood aspirated from patients or healthy controls were separated using Vacutainer mononuclear Cpt SCT (upper left panel). The intermediate phase (monocytic fraction) was extracted by centrifugation as indicated by red letters, and centrifuged cells were visually confirmed by Giemsa staining and phase contrast microscopy (right panel). The protocol for the preparation of protein lysate for Western blotting is summarized in the lower left panel.
