# Supplementary material for: Identification of a Biallelic Missense Variant in Gasdermin D (c.823G > C, p.Asp275His) in a Patient of Atypical Gorham‐Stout Disease in a Consanguineous Family
Source: JBMR Plus. 2023 Jun 29;7(9):e10784. doi: 10.1002/jbm4.10784 (PMC10494506; doi:10.1002/jbm4.10784)
Supplement: Supplementary file 1 — Fig. S1. Clinical outcome with time‐course changes in the osteolytic defect of the fourth distal phalanx of the right hand indicated by plain radiograms (A–H). Radiograms were captured at the first visit in 2014 (A, C), 1 year later (D), 2 years later (E), 3 years later when the denosumab treatment was started (F), and 1 and 2 years after the denosumab treatment (G, H, respectively). Fig. S2. (A) Schematic representation of GSDMD variant 1 (NM_024736.7). Light gray boxes represent coding exons, whereas dark gray boxes represent untranslated regions. The red arrow points to the position of c.823G > C in the exon‐intron junction of exon 7. The black arrows depict the two primer sets used for RT‐PCR. F = forward; R = reverse. (B) RT‐PCR in cDNAs derived from the LCLs of the patient and two controls using two different sets of primers flanking exon 7 of GSDMD. If c.823G > C alters splicing in patient LCLs, a product that is 87 bp shorter may represent the skipping of exon 7, whereas a product that is 130 bp longer indicates the retention of intron 7. Nevertheless, an RT‐PCR product with the same size as those in controls was observed. (C) Partial electropherograms of the Sanger sequencing of RT‐PCR products using primer pair 1 confirmed no skipping of exon 7 or the retention of intron 7 in the patient (bottom), similar to control (top). The position of the c.823G > C variant is highlighted with a rectangle box. (D) qRT‐PCR of GSDMD performed in the patient and three controls showed no significant differences in mRNA levels. Fig. S3. (A, B) qRT‐PCR of mouse Gsdmd (A) and Gsdme (B) was performed in an osteoclast‐inducing culture from RAW 264.7 cells. Relative expression normalized against Gapdh was standardized against the value on day 0. (C, D) RT‐PCR of mouse Gsdmd (C) and Gsdme (D) was performed in an osteoclast‐inducing culture from bone marrow–derived macrophages. Relative expression normalized against Gapdh was further standardized against the value on day 0. Data a [file JBM4-7-e10784-s001.pptx]

## Slide 1
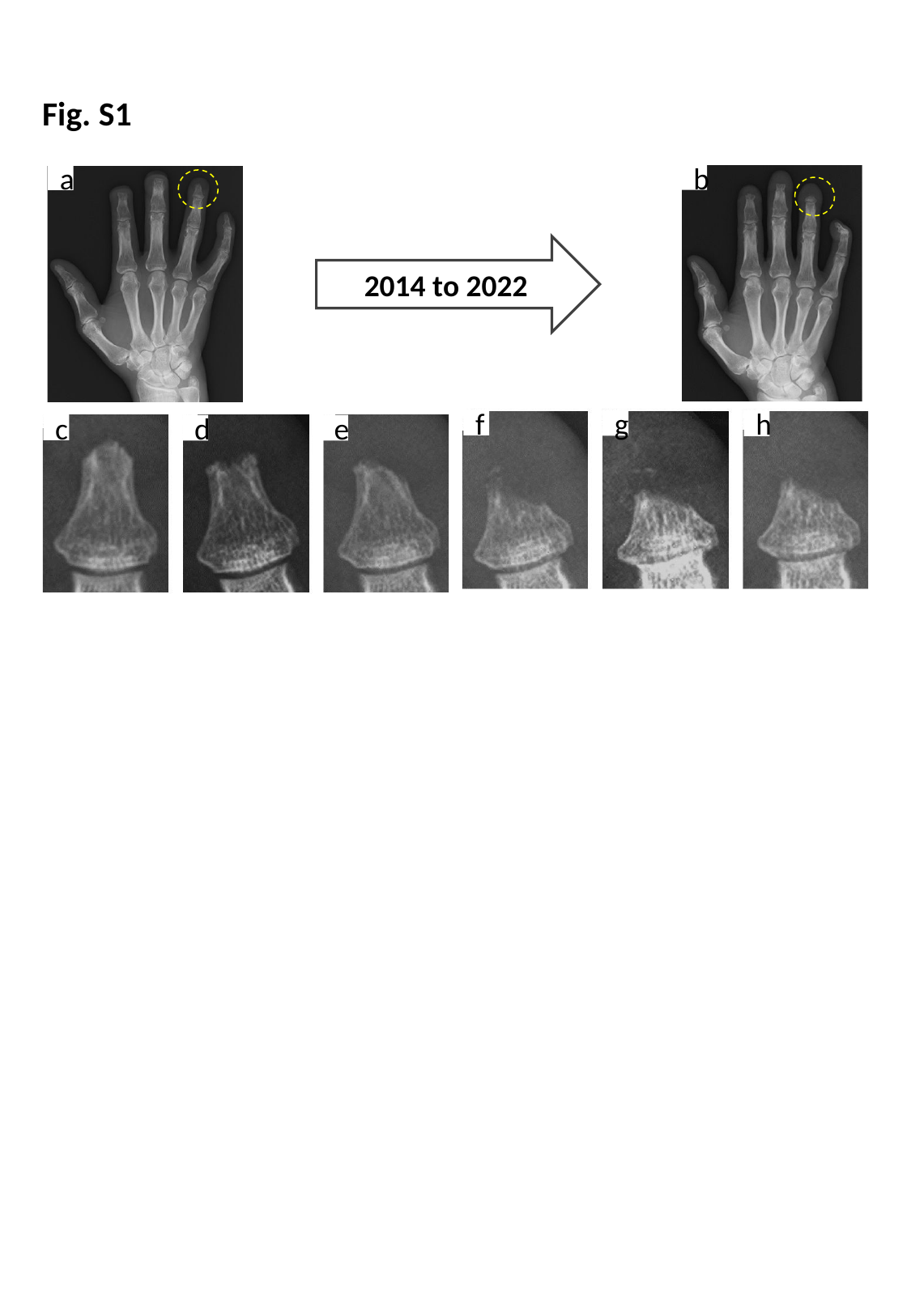

Fig. S1
b
a
f
g
h
c
d
e
2014 to 2022

## Slide 2
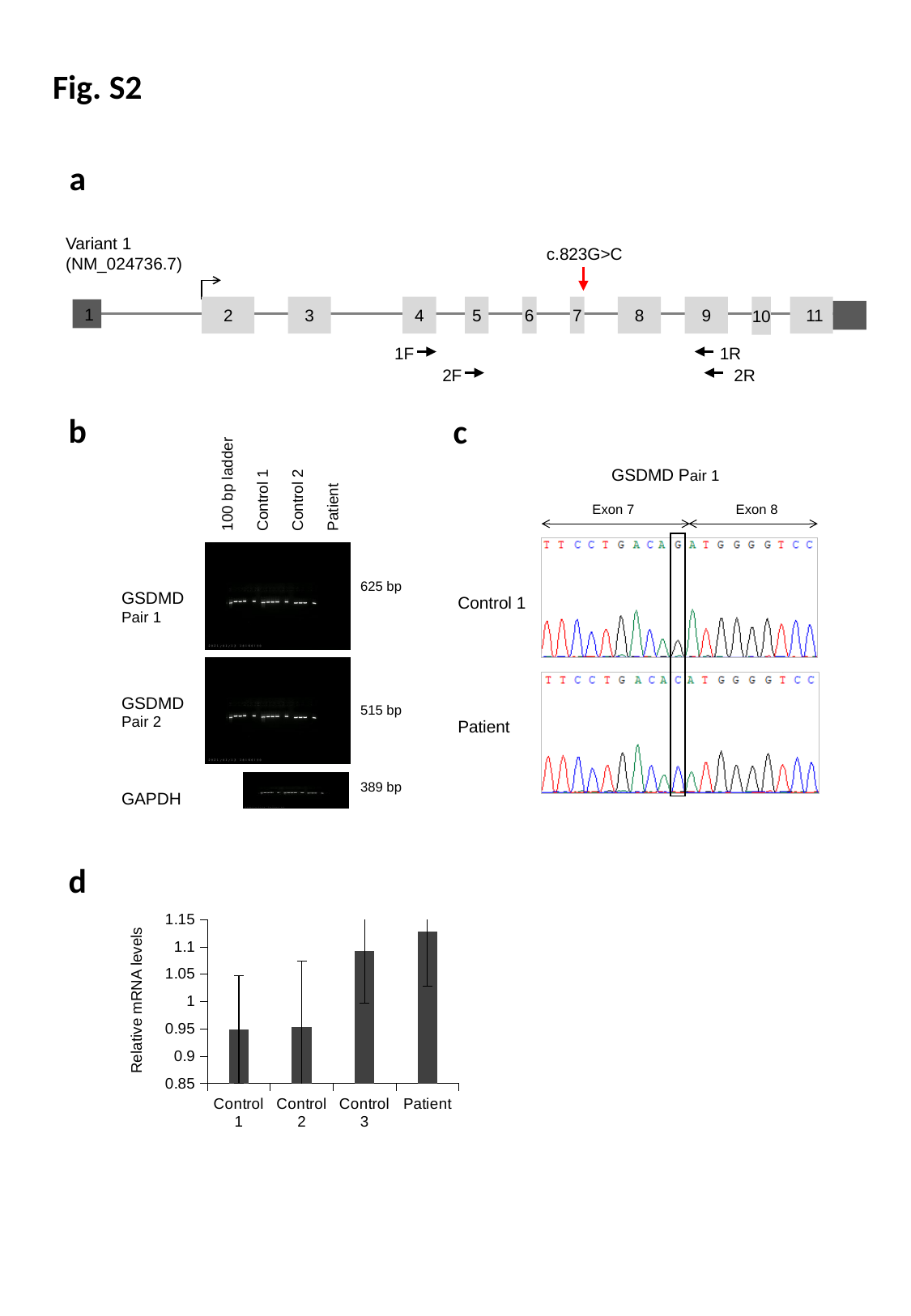

Fig. S2
a
Variant 1 (NM_024736.7)
c.823G>C
2
3
4
5
6
7
8
9
10
11
1
1F
1R
2F
2R
b
c
100 bp ladder
Control 1
Control 2
Patient
GSDMD Pair 1
Exon 7
Exon 8
625 bp
GSDMD Pair 1
Control 1
GSDMD Pair 2
515 bp
Patient
389 bp
GAPDH
d
### Chart
| Category | |
|---|---|
| Control 1 | 0.9486392324880222 |
| Control 2 | 0.9533933335816829 |
| Control 3 | 1.0921968260813435 |
| Patient | 1.128911071431424 |Relative mRNA levels

## Slide 3
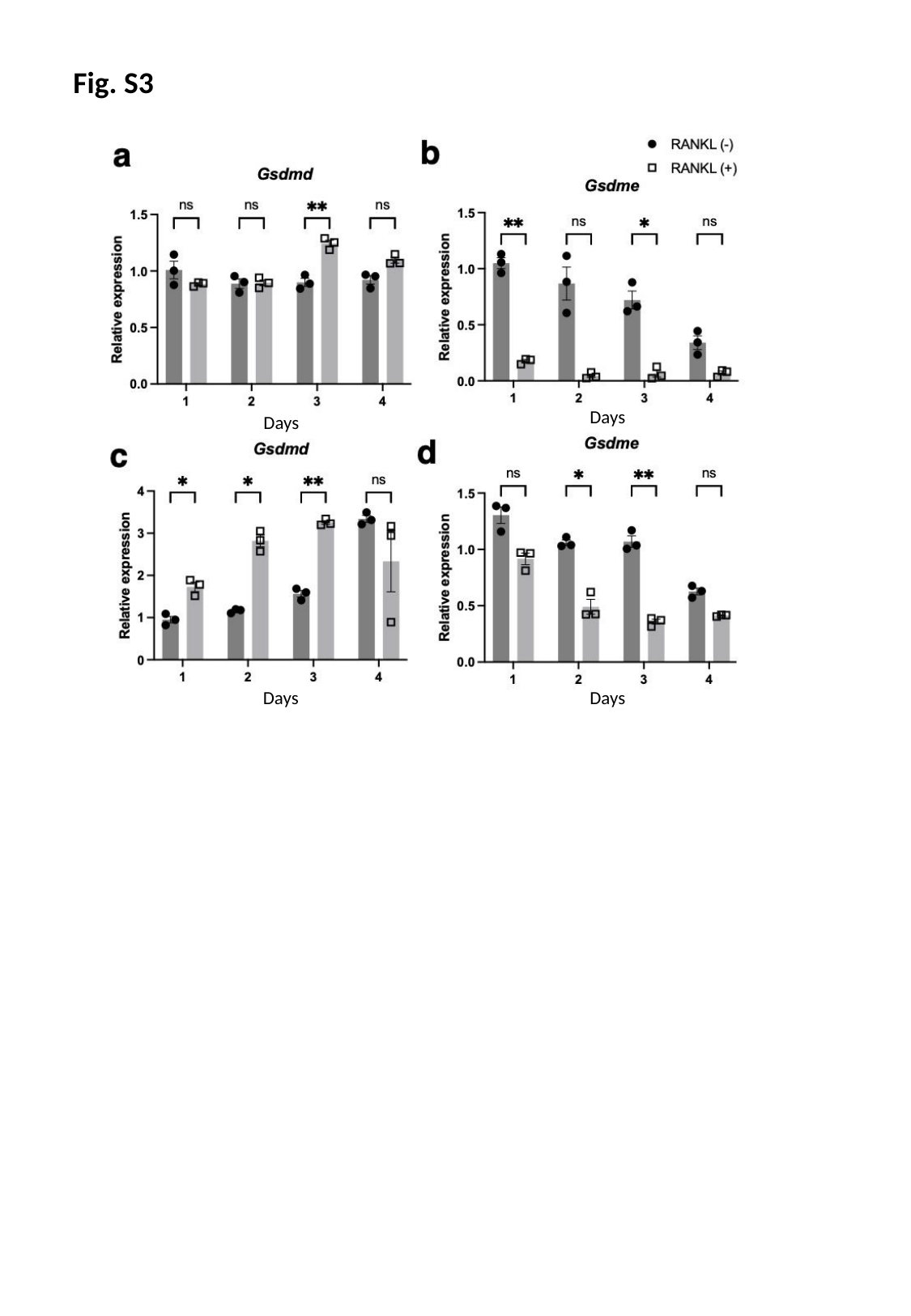

Fig. S3
Days
Days
Days
Days

## Slide 4
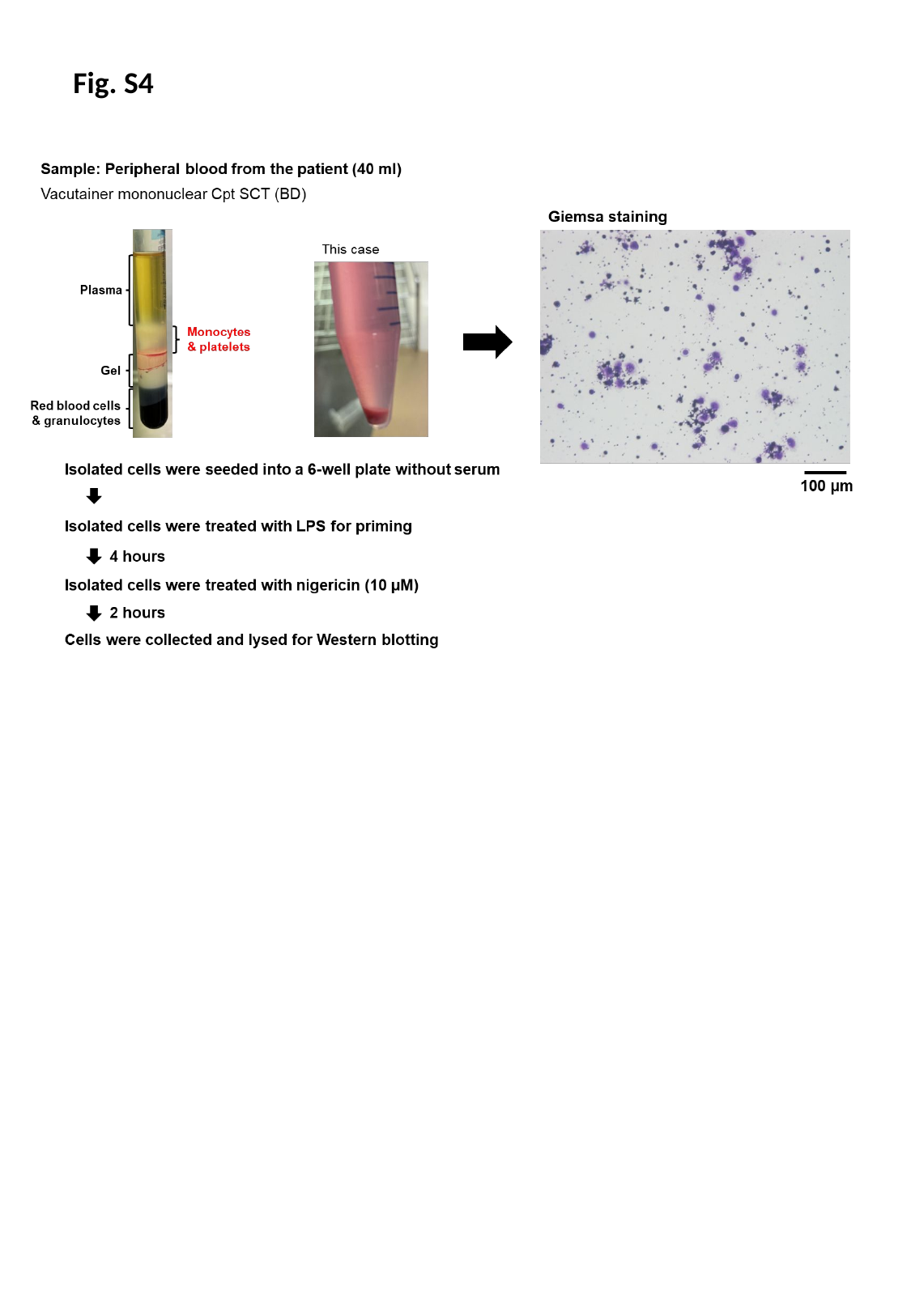

Fig. S4
